# Construction method for three-dimensional dynamic configuration of reciprocating sucker rod string in actual wellbore

**DOI:** 10.1038/s41598-026-52708-z

**Published:** 2026-05-26

**Authors:** Ruidong Zhao, Gaoqiang Ma, Junfeng Shi, Deli Jia, Wenchang Wang, Fang Lan, XiShun Zhang

**Affiliations:** 1 Research Institute of Petroleum Exploration and Development, CNPC Key Laboratory of Oil and Gas Recovery, Beijing City, 100083 China; 2 Research Institute of Oil Production Technology, Yumen Oilfield Branch, CNPC, Jiuquan City, Gansu Province China; 3 School of Mechanics and Engineering Science, Shanghai University, Shanghai City, 200072 China

**Keywords:** Sucker rod string, Dynamics, Buckling, Smooth connection condition, Three-dimensional configuration, Engineering, Mathematics and computing, Physics

## Abstract

The sucker rod string—serving as the core component of rod-pumped oil production systems—exhibits an ultra-slender geometric configuration. Under downhole reciprocating motion, it is prone to complex three-dimensional spatial deformation and may come into contact with the tubing wall, thereby inducing eccentric wear or, in severe cases, mechanical failure such as fracture. Since the dynamic spatial configuration of sucker rod strings is essential for understanding and mitigating tubing–rod eccentric wear, this study proposes a method to construct its three-dimensional spatial configuration under reciprocating movement. The method is grounded in a three-dimensional coupled dynamic model of the sucker rod string within tubing, incorporates buckling stability theory, and introduces smooth transition conditions that ensure deformation continuity of sucker rod string across distinct buckling modes. Finally, engineering case studies are employed to analyze the evolution of the sucker rod string’s three-dimensional spatial configuration within the tubing across distinct phases of reciprocating motion; moreover, the pivotal role of dynamic bottomhole pressure in triggering buckling mode transitions is elucidated.

## Introduction

The sucker rod string exhibits an extremely high aspect ratio (typically > 10⁵), resulting in significant flexibility within the confines of the tubing. During downhole operation—particularly in the downstroke phase when compressive loads act at the bottom—the rod string tends to bend and may even buckle. Given the limited annular space, such deformation causes contact and eccentric wear between rod string and tubing. This is a common problem in rod-pumped wells, eccentric wear often induces sucker rod breakage and disconnection, and increasing the frequency of pump repairs. These issues reduce production efficiency and raise operating costs. With increasing well depth, the slender rod string becomes more susceptible to buckling, further aggravating wear. To mitigate rod-tubing eccentric wear and develop effective countermeasures, it is essential to understand the dynamic spatial configuration of the entire rod string within the tubing. Based on the triaxial coupled mechanical characteristics and buckling critical criterion of sucker rod strings, this paper proposes a method for constructing the three-dimensional configuration of ultra-slender sucker rod strings within tubing under various motion states.

The prerequisite for investigating the spatial configuration of sucker rod string lies in the accurate calculation of its dynamic three-dimensional loads. Early research on sucker rod string was mainly about its axial motion^1,2^. Subsequently, an increasing number of influencing factors were incorporated into the analysis of rod string axial movement, including liquid friction^3,4^, tubing anchoring status^5,6^, rod-tubing-fluid coupling interactions^7,8^, and the effects of centralizers^9,10^. With the widespread application of directional wells, the significance of three-dimensional wellbore trajectories has become increasingly prominent. Xu Jun et al. developed a three-dimensional quasi-static equilibrium equation for sucker rod pumping systems that accounts for well inclination and azimuth, which is applicable to the quasi-static mechanical analysis of low-stroke sucker rod systems^11^. Based on the finite element method, Wang et al. conducted an in-depth investigation into the force and deformation characteristics of sucker rod string, incorporating considerations of the spatial deflection characteristics of actual wellbore and the dual nonlinearity of sucker rod string^12^.

With the increasing complexity of exploitation conditions, the buckling issue of tubing and rod strings has attracted the attention of researchers and engineers. The earliest study on the buckling configuration of tubular string was the helical buckling model for tubing in vertical wells proposed by Lubinski^13^, based on the principle of minimum potential energy, which established the fundamental framework for buckling analysis of petroleum tubular string. With the advancement of drilling technologies, complex structural wells (e.g., deviated wells, directional wells) have become increasingly prevalent. Corresponding research on the critical buckling force of tubular and rod string under diverse wellbore trajectory conditions has been intensively conducted, and the associated theories and methodologies have gradually matured. The stabilizing effect of tubing weight in deviated wells^14^ and the influence of wellbore curvature on force equilibrium^15^ were incorporated into the derivation of the critical load formula. Miska et al. further integrated the torque effect induced by wellbore deflection into the model and revised the calculations of helical pitch and contact force^16^. In the research on critical buckling force, the evolution of buckling modes has long been a focus of scholarly interest. Chen et al. proposed that the critical load for helical buckling is $\:\sqrt{2}$ times that for sinusoidal buckling^17^. Through numerical analysis of the buckling differential equation, Michell derived the critical conditions for the occurrence of helical buckling in sucker rod string during loading and unloading processes^18^.

With advances in research, the focus of studies on buckling has shifted toward more specificity. Mitchell revealed that variations in radial clearance induce oscillations in the helical pitch^19,20^. And the emergence of helices has been considered as a result of localized buckling by some researchers^21,22^. Gao investigated the effect of connectors on the critical buckling force^23^. Miller et al. conducted an experimental investigation into the buckling behavior of slender rods constrained by tubular columns, and revealed the influence of tube-rod clearance and variations in constraint characteristics on the buckling properties of the rod columns^24^.

However, the mechanisms underlying the three-dimensional evolution of the post-buckling configuration, especially the transitions between sinusoidal and helical shapes and their reversibility, continue to be elusive. In response to this, Huang conducted a detailed analysis of the evolution pathway of tubular string buckling in vertical wellbores, encompassing the modes of no buckling, 2D lateral buckling, 3D lateral buckling, continuous contact buckling, and helical buckling. Additionally, they discussed the axial force and torque transfer along the tubular string under the coupling effect of friction and buckling^25^. Building upon this foundation, Liu et al. further integrated theoretical analysis, numerical simulation, and experimental measurements to reveal that under uniaxial compression, a long straight rod first undergoes sinusoidal buckling, followed by snap-through instability leading to the formation of helical buckling. In contrast, during unloading, the helix gradually reverts to a sinusoidal configuration, exhibiting a distinctively differential morphological evolution^26^. Then, Zhang focused on horizontal wells and investigated the buckling transition behavior of compressed tubing string in horizontal wells by integrating the finite element method^27^.

Existing studies have made significant progress in calculating critical buckling loads and characterizing the buckling morphology of tubular strings. Nevertheless, these efforts remain largely confined to idealized, theoretical treatments of localized buckling phenomena; to date, no work has systematically investigated the dynamic three-dimensional spatial configuration of the entire sucker rod string under realistic wellbore conditions. However, In practical engineering applications, eccentric wear of sucker rod strings remains a recurring issue—one that has grown increasingly prominent with the rising number of deep and ultra-deep wells. This phenomenon is primarily attributed to the escalating complexity of wellbore trajectories in such wells, which introduces greater diversity in the mechanical loading states of the rod strings. In turn, this leads to more intricate dynamic deformation behaviors during the rod strings downhole reciprocating motion, as well as heightened uncertainty in their contact interactions with the tubing. Therefore, it is imperative to conduct in-depth investigations into the dynamic three-dimensional spatial configuration of the entire sucker rod string under the complex wellbore conditions of deep and ultra-deep wells.

In this paper, based on the accurate calculation of the three-dimensional force and deformation characteristics of sucker rod strings, and integrating the buckling stability of ultra-long slender rods, we propose a continuity condition for the buckling morphology of ultra-long slender sucker rod strings, and establish a construction method for the dynamic spatial configuration of the entire sucker rod string in actual wellbores. This provides valuable references for understanding and characterizing the dynamic configuration of rod strings within tubing, predicting tubing–rod eccentric wear, and optimizing the structural design of rod strings.

## Theoretical model

### Dynamic model and numerical solution method

**Fig. 1:**
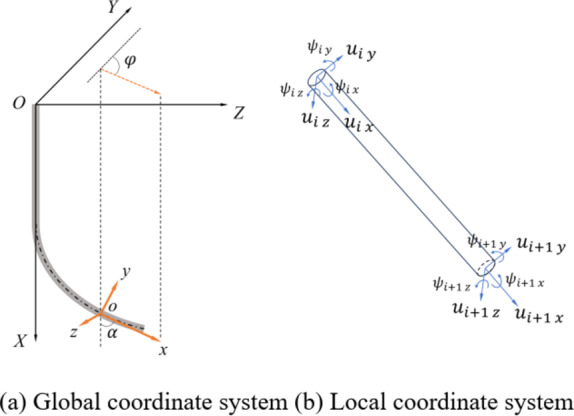
Two coordinate system. (**a**) Global coordinate system, (**b**) Local coordinate system.

The sucker rod string, characterized by an extremely high slenderness ratio, is a flexible slender rod. The motion of such a flexible slender sucker rod string can be decomposed into two components: one is the rigid-body motion synchronized with the polished rod, and the other is the local deformation. To precisely characterize the kinematic and deformation behaviors involved, a global coordinate system (i.e., geographic coordinate system) *O-XYZ* and a local coordinate system *o-xyz* for the element are established for analytical description. The global coordinate system is defined with the wellhead as the origin, where the positive direction of the *X*-axis aligns with the gravitational direction, and the *Y*-axis points to the north. The origin of the local coordinate system is translated along the wellbore axis, where the *x*-axis is oriented tangentially to the wellbore axis toward the well bottom, and the *y*-axis points to the high side of the wellbore, i.e., the principal normal direction, as shown in Fig. 1.

The sucker rod string reciprocates within the tubing, based on the Hamilton’s principle and Lagrange equation, a three-dimensional dynamic finite element model of the sucker rod string can be derived^12,27^.

1 $$\:\boldsymbol{M}\ddot{\boldsymbol{U}}\left(t\right)+\boldsymbol{C}\left(t\right)\dot{\boldsymbol{U}}\left(t\right)+\boldsymbol{K}\boldsymbol{U}\left(t\right)=\boldsymbol{F}\left(t\right)$$

Where, $\:\ddot{\boldsymbol{U}}$, $\:\dot{\boldsymbol{U}}$, $\:\boldsymbol{U}$ are generalized acceleration, velocity and displacement vectors on the nodes of sucker-rod string, each node has six degrees of freedom: three translations along the orthogonal axes and three rotations about them, $\:{u}_{x}$, $\:{u}_{y}$, $\:{u}_{z}$, $\:{\psi\:}_{x}$, $\:{\psi\:}_{y}$, $\:{\psi\:}_{z}$. $\:\boldsymbol{M}$, $\:\boldsymbol{C}$, $\:\boldsymbol{K}$, and $\:\boldsymbol{F}$ denote the mass matrix, damping matrix, stiffness matrix, and nodal load vector of sucker rod, respectively. In the local coordinate system, the element mass matrix takes the form of:

2 $$\:\boldsymbol{M}=\rho\:Al\left[\begin{array}{cccccccccccc}\frac{1}{3}&\:0&\:0&\:0&\:0&\:0&\:\frac{1}{6}&\:0&\:0&\:0&\:0&\:0\\\:0&\:\frac{13}{35}&\:0&\:0&\:0&\:\frac{11l}{210}&\:0&\:\frac{9}{70}&\:0&\:0&\:0&\:-\frac{13l}{420}\\\:0&\:0&\:\frac{13}{35}&\:0&\:-\frac{11l}{210}&\:0&\:0&\:0&\:\frac{9}{70}&\:0&\:\frac{13l}{420}&\:0\\\:0&\:0&\:0&\:\frac{1}{3}\frac{{I}_{x}}{A}&\:0&\:0&\:0&\:0&\:0&\:\frac{1}{6}\frac{{I}_{x}}{A}&\:0&\:0\\\:0&\:0&\:-\frac{11l}{210}&\:0&\:\frac{{l}^{2}}{105}&\:0&\:0&\:0&\:-\frac{13l}{420}&\:0&\:-\frac{{l}^{2}}{140}&\:0\\\:0&\:\frac{11l}{210}&\:0&\:0&\:0&\:\frac{{l}^{2}}{105}&\:0&\:\frac{13l}{420}&\:0&\:0&\:0&\:-\frac{{l}^{2}}{140}\\\:\frac{1}{6}&\:0&\:0&\:0&\:0&\:0&\:\frac{1}{3}&\:0&\:0&\:0&\:0&\:0\\\:0&\:\frac{9}{70}&\:0&\:0&\:0&\:\frac{13l}{420}&\:0&\:\frac{13}{35}&\:0&\:0&\:0&\:-\frac{11l}{210}\\\:0&\:0&\:\frac{9}{70}&\:0&\:-\frac{13l}{420}&\:0&\:0&\:0&\:\frac{13}{35}&\:0&\:\frac{11l}{210}&\:0\\\:0&\:0&\:0&\:\frac{1}{6}\frac{{I}_{x}}{A}&\:0&\:0&\:0&\:0&\:0&\:\frac{1}{3}\frac{{I}_{x}}{A}&\:0&\:0\\\:0&\:0&\:\frac{13l}{420}&\:0&\:-\frac{{l}^{2}}{140}&\:0&\:0&\:0&\:\frac{11l}{210}&\:0&\:\frac{{l}^{2}}{105}&\:0\\\:0&\:-\frac{13l}{420}&\:0&\:0&\:0&\:-\frac{{l}^{2}}{140}&\:0&\:-\frac{11l}{210}&\:0&\:0&\:0&\:\frac{{l}^{2}}{105}\end{array}\right]$$

Where, $\:\rho\:$ denotes the density of the rod string, kg/m³; *\:A* denotes the cross-sectional area of the rod string, m²; *\:l* denotes the length of the rod string element, m; and $\:{I}_{x}$ denotes the polar moment of inertia of the cross-section of the rod string element, m^4^. The element stiffness matrix can be decomposed into a linear stiffness matrix and a nonlinear stiffness matrix, i.e., $\:\boldsymbol{K}={\boldsymbol{K}}_{L}+{\boldsymbol{K}}_{N}$ **.** The linear stiffness matrix $\:{\boldsymbol{K}}_{L}$ takes the form of:

3 $$\:{\boldsymbol{K}}_{L}=\left[\begin{array}{cccccccccccc}\frac{EA}{l}&\:0&\:0&\:0&\:0&\:0&\:-\frac{EA}{l}&\:0&\:0&\:0&\:0&\:0\\\:0&\:\frac{12E{I}_{yz}}{{l}^{3}}&\:0&\:0&\:0&\:\frac{6E{I}_{yz}}{{l}^{2}}&\:0&\:-\frac{12E{I}_{yz}}{{l}^{3}}&\:0&\:0&\:0&\:\frac{6E{I}_{yz}}{{l}^{2}}\\\:0&\:0&\:\frac{12E{I}_{yz}}{{l}^{3}}&\:0&\:-\frac{6E{I}_{yz}}{{l}^{2}}&\:0&\:0&\:0&\:-\frac{12E{I}_{yz}}{{l}^{3}}&\:0&\:-\frac{6E{I}_{yz}}{{l}^{2}}&\:0\\\:0&\:0&\:0&\:\frac{G{I}_{x}}{l}&\:0&\:0&\:0&\:0&\:0&\:-\frac{G{I}_{x}}{l}&\:0&\:0\\\:0&\:0&\:-\frac{6E{I}_{yz}}{{l}^{2}}&\:0&\:\frac{4E{I}_{yz}}{l}&\:0&\:0&\:0&\:\frac{6E{I}_{yz}}{{l}^{2}}&\:0&\:\frac{2E{I}_{yz}}{l}&\:0\\\:0&\:\frac{6E{I}_{yz}}{{l}^{2}}&\:0&\:0&\:0&\:\frac{4E{I}_{yz}}{l}&\:0&\:-\frac{6E{I}_{yz}}{{l}^{2}}&\:0&\:0&\:0&\:\frac{2E{I}_{yz}}{l}\\\:-\frac{EA}{l}&\:0&\:0&\:0&\:0&\:0&\:\frac{EA}{l}&\:0&\:0&\:0&\:0&\:0\\\:0&\:-\frac{12E{I}_{yz}}{{l}^{3}}&\:0&\:0&\:0&\:-\frac{6E{I}_{yz}}{{l}^{2}}&\:0&\:\frac{12E{I}_{yz}}{{l}^{3}}&\:0&\:0&\:0&\:-\frac{6E{I}_{yz}}{{l}^{2}}\\\:0&\:0&\:-\frac{12E{I}_{yz}}{{l}^{3}}&\:0&\:\frac{6E{I}_{yz}}{{l}^{2}}&\:0&\:0&\:0&\:\frac{12E{I}_{yz}}{{l}^{3}}&\:0&\:\frac{6E{I}_{yz}}{{l}^{2}}&\:0\\\:0&\:0&\:0&\:-\frac{G{I}_{x}}{l}&\:0&\:0&\:0&\:0&\:0&\:\frac{G{I}_{x}}{l}&\:0&\:0\\\:0&\:0&\:-\frac{6E{I}_{yz}}{{l}^{2}}&\:0&\:\frac{2E{I}_{yz}}{l}&\:0&\:0&\:0&\:\frac{6E{I}_{yz}}{{l}^{2}}&\:0&\:\frac{4E{I}_{yz}}{l}&\:0\\\:0&\:\frac{6E{I}_{yz}}{{l}^{2}}&\:0&\:0&\:0&\:\frac{2E{I}_{yz}}{l}&\:0&\:-\frac{6E{I}_{yz}}{{l}^{2}}&\:0&\:0&\:0&\:\frac{4E{I}_{yz}}{l}\end{array}\right]$$

Where, $\:{I}_{yz}$ denotes the moment of inertia of the cross-section of the drill string element, m⁴; *E* represents the modulus of elasticity, Pa; and *G* stands for the shear modulus, Pa. The nonlinear stiffness matrix $\:{\boldsymbol{K}}_{N}$ can be decomposed into three components, i.e., $\:{\boldsymbol{K}}_{N}={\boldsymbol{K}}_{N1}+{\boldsymbol{K}}_{N2}+{\boldsymbol{K}}_{N3}$, where $\:{\boldsymbol{K}}_{N1}$ denotes the coupling between axial and torsional, $\:{\boldsymbol{K}}_{N2}$ denotes the coupling between axial and lateral, and $\:{\boldsymbol{K}}_{N3}$ denotes the coupling between torsional and lateral. Their expressions are given by:

4 $$\:{\boldsymbol{K}}_{N1}=\frac{EA({u}_{i\:x}-{u}_{i-1\:x})}{{l}^{2}}\left[\begin{array}{cccccccccccc}\frac{3}{2}&\:0&\:0&\:0&\:0&\:0&\:-\frac{3}{2}&\:0&\:0&\:0&\:0&\:0\\\:0&\:\frac{6}{5}&\:0&\:0&\:0&\:\frac{l}{10}&\:0&\:-\frac{6}{5}&\:0&\:0&\:0&\:\frac{l}{10}\\\:0&\:0&\:\frac{6}{5}&\:0&\:-\frac{l}{10}&\:0&\:0&\:0&\:-\frac{6}{5}&\:0&\:-\frac{l}{10}&\:0\\\:0&\:0&\:0&\:\frac{{I}_{x}}{A}&\:0&\:0&\:0&\:0&\:0&\:-\frac{{I}_{x}}{A}&\:0&\:0\\\:0&\:0&\:-\frac{l}{10}&\:0&\:\frac{2{l}^{2}}{15}&\:0&\:0&\:0&\:\frac{l}{10}&\:0&\:-\frac{{l}^{2}}{30}&\:0\\\:0&\:\frac{l}{10}&\:0&\:0&\:0&\:\frac{2{l}^{2}}{15}&\:0&\:-\frac{l}{10}&\:0&\:0&\:0&\:-\frac{{l}^{2}}{30}\\\:-\frac{3}{2}&\:0&\:0&\:0&\:0&\:0&\:\frac{3}{2}&\:0&\:0&\:0&\:0&\:0\\\:0&\:-\frac{6}{5}&\:0&\:0&\:0&\:-\frac{l}{10}&\:0&\:\frac{6}{5}&\:0&\:0&\:0&\:-\frac{l}{10}\\\:0&\:0&\:-\frac{6}{5}&\:0&\:\frac{l}{10}&\:0&\:0&\:0&\:\frac{6}{5}&\:0&\:\frac{l}{10}&\:0\\\:0&\:0&\:0&\:-\frac{{I}_{x}}{A}&\:0&\:0&\:0&\:0&\:0&\:\frac{{I}_{x}}{A}&\:0&\:0\\\:0&\:0&\:-\frac{l}{10}&\:0&\:-\frac{{l}^{2}}{30}&\:0&\:0&\:0&\:\frac{l}{10}&\:0&\:\frac{2{l}^{2}}{15}&\:0\\\:0&\:\frac{l}{10}&\:0&\:0&\:0&\:-\frac{{l}^{2}}{30}&\:0&\:-\frac{l}{10}&\:0&\:0&\:0&\:\frac{2{l}^{2}}{15}\end{array}\right]$$

5 $$\:{\boldsymbol{K}}_{N2}=\frac{E{I}_{yz}({u}_{i\:x}-{u}_{i-1\:x})}{{l}^{4}}\left[\begin{array}{cccccccccccc}0&\:0&\:0&\:0&\:0&\:0&\:0&\:0&\:0&\:0&\:0&\:0\\\:0&\:36&\:0&\:0&\:0&\:18l&\:0&\:-36&\:0&\:0&\:0&\:18l\\\:0&\:0&\:36&\:0&\:-18l&\:0&\:0&\:0&\:-36&\:0&\:-18l&\:0\\\:0&\:0&\:0&\:0&\:0&\:0&\:0&\:0&\:0&\:0&\:0&\:0\\\:0&\:0&\:-18l&\:0&\:12{l}^{2}&\:0&\:0&\:0&\:18l&\:0&\:6{l}^{2}&\:0\\\:0&\:18l&\:0&\:0&\:0&\:12{l}^{2}&\:0&\:-18l&\:0&\:0&\:0&\:6{l}^{2}\\\:0&\:0&\:0&\:0&\:0&\:0&\:0&\:0&\:0&\:0&\:0&\:0\\\:0&\:-36&\:0&\:0&\:0&\:-18l&\:0&\:36&\:0&\:0&\:0&\:-18l\\\:0&\:0&\:-36&\:0&\:18l&\:0&\:0&\:0&\:36&\:0&\:18l&\:0\\\:0&\:0&\:0&\:0&\:0&\:0&\:0&\:0&\:0&\:0&\:0&\:0\\\:0&\:0&\:-18l&\:0&\:6{l}^{2}&\:0&\:0&\:0&\:18l&\:0&\:12{l}^{2}&\:0\\\:0&\:18l&\:0&\:0&\:0&\:6{l}^{2}&\:0&\:-18l&\:0&\:0&\:0&\:12{l}^{2}\end{array}\right]$$

6 $$\:{\boldsymbol{K}}_{N3}=\frac{(1+2\upsilon\:)G{I}_{x}({\psi\:}_{i\:x}-{\psi\:}_{i-1\:x})}{{l}^{2}}\left[\begin{array}{cccccccccccc}0&\:0&\:0&\:\frac{1+\upsilon\:}{1+2\upsilon\:}&\:0&\:0&\:0&\:0&\:0&\:-\frac{1+\upsilon\:}{1+2\upsilon\:}&\:0&\:0\\\:0&\:0&\:0&\:0&\:-1&\:0&\:0&\:0&\:0&\:0&\:1&\:0\\\:0&\:0&\:0&\:0&\:0&\:-1&\:0&\:0&\:0&\:0&\:0&\:1\\\:0&\:0&\:0&\:0&\:0&\:0&\:0&\:0&\:0&\:0&\:0&\:0\\\:0&\:-1&\:0&\:0&\:0&\:0&\:0&\:1&\:0&\:0&\:0&\:-\frac{l}{2}\\\:0&\:0&\:-1&\:0&\:0&\:0&\:0&\:0&\:1&\:0&\:\frac{l}{2}&\:0\\\:0&\:0&\:0&\:-\frac{1+\upsilon\:}{1+2\upsilon\:}&\:0&\:0&\:0&\:0&\:0&\:\frac{1+\upsilon\:}{1+2\upsilon\:}&\:0&\:0\\\:0&\:0&\:0&\:0&\:1&\:0&\:0&\:0&\:0&\:0&\:-1&\:0\\\:0&\:0&\:0&\:0&\:0&\:1&\:0&\:0&\:0&\:0&\:0&\:-1\\\:0&\:0&\:0&\:0&\:0&\:0&\:0&\:0&\:0&\:0&\:0&\:0\\\:0&\:1&\:0&\:0&\:0&\:\frac{l}{2}&\:0&\:-1&\:0&\:0&\:0&\:0\\\:0&\:0&\:1&\:0&\:-\frac{l}{2}&\:0&\:0&\:0&\:-1&\:0&\:0&\:0\end{array}\right]$$

Where, $\:\upsilon\:$ denotes Poisson’s ratio.

During the motion of the sucker rod, the damping effect of downhole fluid is a critical factor that is both non-negligible and challenging to address. Currently, the Rayleigh damping model is predominantly employed for simplification, whose essence lies in expressing the damping acting on the sucker rod string (including structural damping and drilling fluid damping) as a linear combination of the stiffness matrix and the mass matrix.

Once the upper dynamometer card near the surface and the rod string load data near the pump have been measured on site, the damping coefficient can be fitted based on the loads at the upper and lower ends. The expression of the damping matrix $\:\boldsymbol{C}$ is as follows^28^:

7 $$\:C=\alpha\:M+\beta\:K$$

Where, $\:\alpha\:$ and $\:\beta\:$ are damping coefficients, which can be determined by the natural frequencies of the system and their corresponding damping ratios.

The equivalent nodal force due to gravity is:

8 $$\:{\boldsymbol{F}}_{g}=\left[\begin{array}{cccccccccccc}\frac{{q}_{x}l}{2}&\:\frac{{q}_{y}l}{2}&\:0&\:0&\:0&\:\frac{{q}_{y}{l}^{2}}{12}&\:\frac{{q}_{x}l}{2}&\:\frac{{q}_{y}l}{2}&\:0&\:0&\:0&\:-\frac{{q}_{y}{l}^{2}}{12}\end{array}\right]$$

Where, $\:{q}_{x}={q}_{e}{cos}\alpha\:$, $\:{q}_{y}={q}_{e}{sin}\alpha\:$。$\:{q}_{e}$ represents the unit buoyancy force, N; $\:\alpha\:$ is the well deviation angle, rad. For actual calculations, the measured dynamic concentrated force at the end of the sucker rod string should also be superimposed on the nodal load matrix.

When solving the dynamic finite element matrix Eq. (1), the boundary conditions can be adopted as constrained displacement at the bottom end and time-varying tensile force at the upper end given by the dynamometer card. So the elastic deformation is first calculated numerically, and then superimposed with the rigid motion determined by the motion law of the suspension point of pumping unit, thereby obtaining the total displacement of the sucker rod string. Based on the successive over-relaxation (SOR) nodal iterative method and the Newmark time integration method, the dynamic finite element model can be written as^28^:

9 $$\:{\stackrel{\sim}{\boldsymbol{K}}}_{t+\varDelta\:t}{\boldsymbol{U}}_{t+\varDelta\:t}={\stackrel{\sim}{\boldsymbol{F}}}_{t+\varDelta\:t}$$

where $\:{\stackrel{\sim}{\boldsymbol{K}}}_{t+\varDelta\:t}$ is the equivalent stiffness matrix, $\:{\stackrel{\sim}{\boldsymbol{F}}}_{t+\varDelta\:t}$ is the equivalent force matrix, and can be expressed as:

10 $$\:{\stackrel{\sim}{K}}_{t+\varDelta\:t}={\lambda\:}_{1}M+{\lambda\:}_{4}C+K$$

11 $$\:{\stackrel{\sim}{\boldsymbol{F}}}_{t+\varDelta\:t}={\boldsymbol{F}}_{t+\varDelta\:t}+\boldsymbol{M}\left({\lambda\:}_{1}{\boldsymbol{U}}_{t}+{\lambda\:}_{2}{\dot{\boldsymbol{U}}}_{t}+{\lambda\:}_{3}{\ddot{\boldsymbol{U}}}_{t}\right)+\boldsymbol{C}\left({\lambda\:}_{4}{\boldsymbol{U}}_{t}+{\lambda\:}_{5}{\dot{\boldsymbol{U}}}_{t}+{\lambda\:}_{6}{\ddot{\boldsymbol{U}}}_{t}\right)$$

Where, $\:{\lambda\:}_{i}$ (i = 1,2,…6) is the weighting coefficient specified by the Newmark method. When using the SOR node iteration, each element $\:{u}_{i}$ in the vector $\:\boldsymbol{U}$ can be expressed as:

$$\:{\boldsymbol{u}}_{i}^{\left(k+1\right)}={\boldsymbol{u}}_{i}^{\left(k\right)}+\frac{\omega\:}{{a}_{ii}}\left({b}_{i}-{\sum\:}_{j=1}^{i-1}{a}_{ij}{\boldsymbol{u}}_{j}^{\left(k+1\right)}-{\sum\:}_{j=i}^{n}{a}_{ij}{\boldsymbol{u}}_{j}^{\left(k\right)}\right)$$

Where, $\:{a}_{ii}$ and $\:{a}_{ij}$ are corresponding elements in matrix $\:\stackrel{\sim}{\boldsymbol{K}}$, $\:{b}_{i}$ is corresponding element in matrix $\:\stackrel{\sim}{\boldsymbol{F}}$. Superscript *k* denotes the *k*-th iteration. *ω* is the relaxation factor, when 1≤$\:\omega\:$<2, the correction term is amplified, thereby accelerating convergence.

If the contact between sucker rod string and tubing occurred, the nodal loads at the corresponding positions can be corrected using the Hertz contact model^29^:

12 $$\:{F}_{yz}=\left\{\begin{array}{l}0\:\:\:\:\:\:\:\:\:\:\:\:\:\:\:\:\:\:\:\:\:\:\:\:\:\:\:\:\:\:\:\:\:\:\:\:\:\:\:\:\:\:\:\:{u}_{yz}\le\:R-r\\\:{K}_{h}{\left({u}_{yz}+R-r\right)}^{3/2}\:\:\:\:\:\:\:{\:\:u}_{yz}>R-r\end{array}\right.$$

13 $$\:{F}_{x}={\mu\:}_{h}{F}_{yz}\mathrm{s}\mathrm{g}\mathrm{n}\left({\dot{u}}_{x}\right)$$

Where, $\:{F}_{yz}$ denotes the contact force, N; $\:{F}_{x}$ denotes the frictional force induced by contact, N; $\:{u}_{yz}$ is the radial displacement, m; *\:R* and *\:r* are respectively the inner radius of the tubing and the outer radius of the sucker rod string, m; $\:{\mu\:}_{h}$ is the friction coefficient between the sucker rod string and the tubing, dimensionless. Since the contact positions and deformations of the sucker rod string at each moment tend to stabilize through iterative cycles, such frictional resistance finally converges to a steady value in the iterative calculation at each time step.

### Buckling model and its solution methodology

On the basis of calculating the load and deformation of the sucker rod string via the three-dimensional coupled dynamic model, the buckling stability of the sucker rod string can be further analyzed. Under the constraint of the outer casing, the buckling behavior of the sucker rod string displays distinctive mechanical characteristics that deviate from classical Euler buckling. Owing to the lateral confinement imposed by the wellbore and tubing on the sucker rod string, progressive axial loading induces a distinct morphological transition: from an initially stable configuration, through sinusoidal buckling, to fully developed helical buckling. Assuming that the sucker rod string maintains continuous contact with the tubing after buckling, the governing differential equation for the buckled sucker rod string can be derived based on equilibrium relationships^19,25^.

14 $$\:\frac{{d}^{4}\theta\:}{d{x}^{4}}-6{\left(\frac{d\theta\:}{dx}\right)}^{2}\frac{{d}^{2}\theta\:}{d{x}^{2}}+3\frac{{M}_{T}}{EI}\frac{d\theta\:}{dx}\frac{{d}^{2}\theta\:}{d{x}^{2}}+\frac{d}{dx}\left(\frac{F}{EI}\frac{d\theta\:}{dx}\right)+\frac{q\mathrm{sin}\alpha\:}{EIr}\mathrm{sin}\theta\:=0$$

**Fig. 2:**
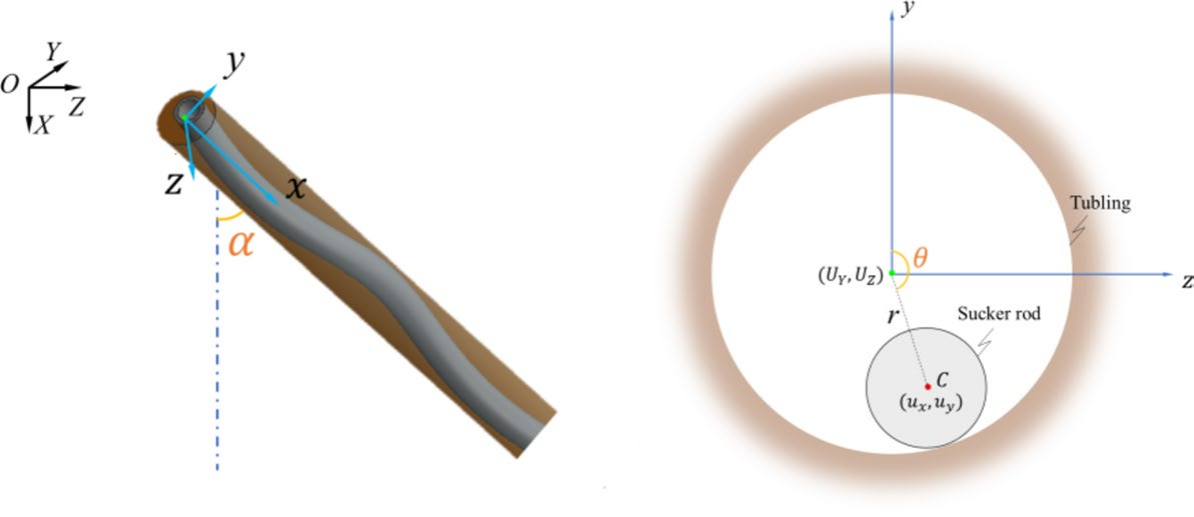
Schematic diagram of the post-buckling position of the sucker rod string within the tubing.

Where, $\:{M}_{T}$ denotes the torque acting on the sucker rod string, N·m; *\:E* represents the elastic modulus of the rod string, Pa; *\:I* is the polar moment of inertia of the rod string’s cross-section, m⁴; *F* denotes the axial load, with units of N, the nonlinear coupled finite element model presented in Sect.  2.1 enables the derivation of a relatively accurate axial load; *\:q* is the buoyed weight per unit length of the sucker rod string, N/m; $\:\alpha\:$ indicates the well inclination angle, rad; $\:\theta\:$ denotes the deflection angle of the sucker rod string relative to its initial position, with units of rad; *\:r* represents the maximum distance between the center of the sucker rod and that of the tubing, m. As illustrated in Fig. 2.

The Galerkin method is employed to solve the buckling differential Eq. (14), yielding the deflection angle function of the pipe string under sinusoidal and helical buckling as follows^30^:

15 $$\:\begin{array}{c}\left\{\begin{array}{c}{\theta\:}_{s}=\sqrt{\frac{8\left[{\left(1-\epsilon\:\xi\:{cos}\alpha\:\right)}^{2}-{Q}_{0}\mathrm{sin}\alpha\:\right]}{12{\left(1-\epsilon\:\xi\:{cos}\alpha\:\right)}^{2}-{Q}_{0}{sin}\alpha\:}}\mathrm{sin}\left(\eta\:\right)\:\:\:\:\:\:\:\:\:\:\:\:\:\:\:sinusoidal\:buckling\\\:{\theta\:}_{h}=\pm\:\left(\eta\:-\frac{1}{5}{Q}_{1}\mathrm{sin}\eta\:-\frac{7}{1600}{Q}_{1}^{2}\mathrm{sin}2\eta\:\right)\:\:\:\:\:\:\:\:\:\:\:\:\:\:\:\:\:\:\:\:\:\:\:\:\:\:helical\:buckling\end{array}\right.\:\:\end{array}$$

Where, $\:{Q}_{0}=\frac{q}{EIr{\omega\:}^{4}}$, $\:{Q}_{1}=\frac{{Q}_{0}\mathrm{sin}\alpha\:}{{\left(1-\epsilon\:\xi\:\mathrm{cos}\alpha\:\right)}^{2}}$, $\:\omega\:=\sqrt{\frac{F}{2EI}}$, $\:\xi\:=\omega\:x$, $\:\eta\:=\frac{2}{3\epsilon\:\mathrm{cos}\alpha\:}\left[1-{\left(1-\epsilon\:\xi\:\mathrm{cos}\alpha\:\right)}^{3/2}\right]$, *ε* is an infinitesimal quantity, denoting the relative rate of change of the axial force along the length of the rod string.

Once the axial compressive force reaches a specific critical value, the pipe string will undergo sinusoidal or helical buckling. The critical buckling forces of the sucker rod string corresponding to different types of well sections could be derived via the energy method shown as follows^20^:

(1) Vertical wellbore Sect. 

16 $$\:{P}_{cs}=1.94(EI{q}^{2}{)}^{\frac{1}{3}}\:\:\:\:\:\:\:\:\:\:\:\:\:\:\:\:\:\:\:\:\:\:\:\:\:\left(\alpha\:<{\alpha\:}_{c}\right)$$

Where, $\:{\alpha\:}_{c}$ is a small quantity, $\:{\alpha\:}_{c}=\mathrm{arcsin}\left[0.94r{\left(\frac{q}{EI}\right)}^{\frac{1}{3}}\right]$.

(2) Deviated straight wellbore Sect. 

17 $$\:{P}_{cs}=2{\left(\frac{EIq\mathrm{sin}\alpha\:}{r}\right)}^{\frac{1}{2}}$$

In the vertical or deviated wellbore section, the critical force for helical buckling is 1.377 times that for sinusoidal buckling.

(3) Curved well section.

The curved section can be categorized into build-up well section and drop-off well section.

(a) Build-up well section:

18 $$\:{P}_{cs}=\frac{2EIk}{r}+2{\left[{\left(\frac{EIk}{r}\right)}^{2}+\frac{EIq\mathrm{sin}\alpha\:}{r}\right]}^{\frac{1}{2}}$$

Where *k* denotes the curvature, m^− 1^. For the build-up well section, the stability of the rod string tends to be enhanced.

(b) Drop-off well section:

19 $$\:{P}_{cs}=\left\{\begin{array}{c}\frac{2EIk}{r}-2{\left[{\left(\frac{EIk}{r}\right)}^{2}-\frac{EIq\mathrm{sin}\alpha\:}{r}\right]}^{\frac{1}{2}}\:\:\:\:\:\:\:\:\:\:\:\:\:\:\:(k\ge\:{k}_{0})\\\:-\frac{2EIk}{r}+2{\left[{\left(\frac{EIk}{r}\right)}^{2}+\frac{EIq\mathrm{sin}\alpha\:}{r}\right]}^{\frac{1}{2}}\:\:\:\:\:\:\:\:\:\:\:(k<{k}_{0})\end{array}\right.$$

Where, $$\:{k}_{0}={\left(\frac{rq\:sin\alpha\:}{EI}\right)}^{\frac{1}{2}}$$ .

For the helical buckling in curved wellbores, Mitchell demonstrated that the critical buckling load depends on the axial load variation: when compressive load increases (usually during the initial phase of the downstroke), the critical helical buckling load is 2.828 times that of sinusoidal buckling; conversely, when compressive load decreases (usually during the initial phase of the upstroke), the critical helical buckling load reduces to 1.414 times that of sinusoidal buckling.

### The smooth connection conditions and three-dimensional configuration

When the sucker rod string undergoes buckling, based on the continuous contact assumption, the lateral deformation of sucker rod string can be described by the parametric equations:

20 $$\:\left\{\begin{array}{c}{u}_{y}=r{cos}\theta\:\\\:{u}_{z}=r{sin}\theta\:\end{array}\right.$$

Once *θ* is solved via Eq. (15), the spatial configuration of the buckled rod string segment can be computed using this equation. However, owing to the distinct deflection angle functions employed for sinusoidal buckling and helical buckling, a configuration discontinuity issue arises at the interface between the sinusoidal buckling segment and the helical buckling segment. In addition, the spatial deformation of the unbuckled rod string segment is computed via a three-dimensional finite element dynamic model, thus also resulting in a jump at the junction of the unbuckled and the buckled segment. To ensure the physical plausibility of the numerical results, the smooth connection condition must be satisfied between the different segments:

21 $$\:{u}_{y\:i}^{{\prime\:}}\left({L}_{i}^{n}\right)={u}_{y\:i+1}^{{\prime\:}}\left({L}_{i+1}^{0}\right)\:,\:\:\:\:\:\:\:\:or\:\:\:\:\:\:\:\:{u}_{z\:i}^{{\prime\:}}\left({L}_{i}^{n}\right)={u}_{z\:i+1}^{{\prime\:}}\left({L}_{i+1}^{0}\right)\:\:\:\:\:\:\:\:\:\:\:\:\:i=\mathrm{1,2}$$

Where, the subscript *i* denotes distinct segments: subscript 1 denotes the unbuckled segment; subscript 2 denotes the sinusoidal buckling segment; subscript 3 denotes the helical buckling segment. The superscripts *0* and *n* respectively represent the sequence number of the first and last nodes within each segment. When the unbuckled segment is connected to the sinusoidal buckling segment, the initial rotation angle of the sinusoidal buckling segment can be determined by considering Eqs. (15), (20), and (21) as follows:

22 $$\:{\widehat{\theta\:}}_{s}=\mathrm{arcsin}\frac{{{u}_{y\:1}\left({L}_{1}^{n-1}\right)-u}_{y\:1}\left({L}_{1}^{n}\right)}{r{\Delta\:}{L}_{1}^{n}}\:,\:\:\:\:\:\:or\:\:\:\:\:\:{\widehat{\theta\:}}_{s}=\mathrm{arccos}\frac{{u}_{z\:1}\left({L}_{1}^{n}\right)-{u}_{z\:1}\left({L}_{1}^{n-1}\right)}{r{\Delta\:}{L}_{1}^{n}}$$

Where, $\:{\Delta\:}{L}_{1}^{n}={L}_{1}^{n}-{L}_{1}^{n-1}$, Then, the actual rotation angles at each point along the sinusoidal buckling segment of the rod is $\:{\stackrel{\sim}{\theta\:}}_{s}={\theta\:}_{s}-({\theta\:}_{s}^{0}-{\widehat{\theta\:}}_{s})$. Similarly, the initial rotation angle of the helical buckling segment is:

23 $$\:{\widehat{\theta\:}}_{h}=\mathrm{arcsin}\frac{{{u}_{y\:2}\left({L}_{2}^{n-1}\right)-u}_{y\:2}\left({L}_{2}^{n}\right)}{r{\Delta\:}{L}_{2}^{n}}\:,\:\:\:\:\:\:or\:\:\:\:\:\:{\widehat{\theta\:}}_{h}=\mathrm{arccos}\frac{{u}_{z\:2}\left({L}_{2}^{n}\right)-{u}_{z\:2}\left({L}_{2}^{n-1}\right)}{r{\Delta\:}{L}_{2}^{n}}$$

So, the actual rotation angles at each point along the helical buckling segment is $\:{\stackrel{\sim}{\theta\:}}_{h}={\theta\:}_{h}-({\theta\:}_{h}^{0}-{\widehat{\theta\:}}_{h})$. That is, the rotation angles of the sinusoidal buckling section and the helical buckling section were successively corrected by rotation to meet the smooth connection conditions.

Based on the three-dimensional dynamic finite element model of the sucker rod string, the spatial deformation of the entire sucker rod string at each moment can be computed via node iteration. Once the iterative calculation of the spatial deformation of the entire rod string at a given moment has converged and stabilized, the load distribution along the string is determined via the stiffness matrix. On this basis, the buckling state of the lower sucker rod string is further assessed by incorporating the wellbore trajectory characteristics and the buckling criterion. If no buckling is detected in the rod string at that moment, the spatial configuration of the entire rod string is taken as the spatial deformation directly calculated by the finite element model. If buckling of the sucker rod string is detected, the sinusoidal or helical buckling mode of the buckled segment is calculated in accordance with Eqs. (15) and (20). Subsequently, the initial phase of the buckling mode is adjusted based on Eqs. (22) and (23) to satisfy the smooth connection conditions (21), ultimately yielding the continuous spatial configuration of the entire sucker rod string at the current moment. That is to say, the calculation converges at each time step, the buckling mode of the sucker rod string at that time step is computed accordingly. Upon completion of calculations for all time steps, the dynamic spatial configurations of the entire sucker rod string across all time steps are constructed. Considering the convergence requirements of the calculation and the gradual nature of buckling mode changes during rod string motion, the effect of buckling mode variations on velocity and acceleration is neglected in the calculation process.

**Fig. 3:**
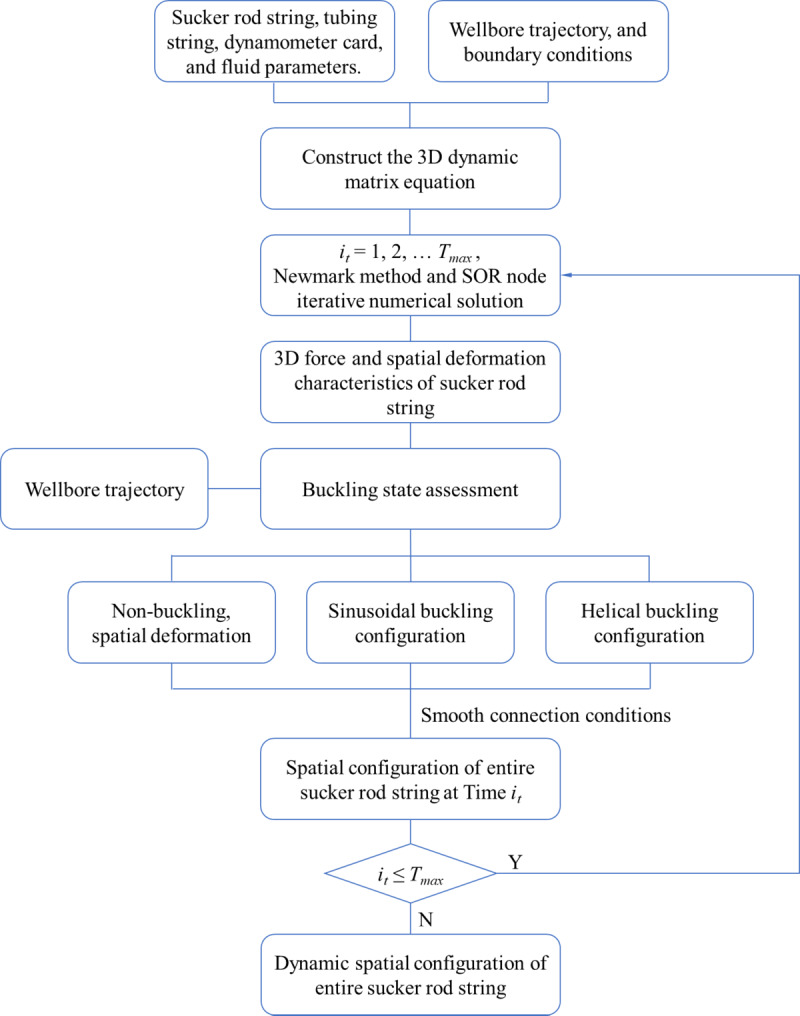
Flowchart for constructing the spatial configuration of the entire sucker rod string.

The calculation process is shown in Fig. 3.

### Verification and validation notes

The methodological workflow proposed in this study can be broadly divided into three core components: calculation of the three-dimensional mechanical characteristics of the sucker rod string, critical buckling criterion validation, and spatial configuration construction. The accuracy of the three-dimensional mechanical characteristic calculation method for the rod string will be verified via comparison with field-measured data in the case study section. In this section, the critical helical buckling force obtained from the proposed method is compared with laboratory test results in the literature to validate its accuracy.

**Fig. 4:**
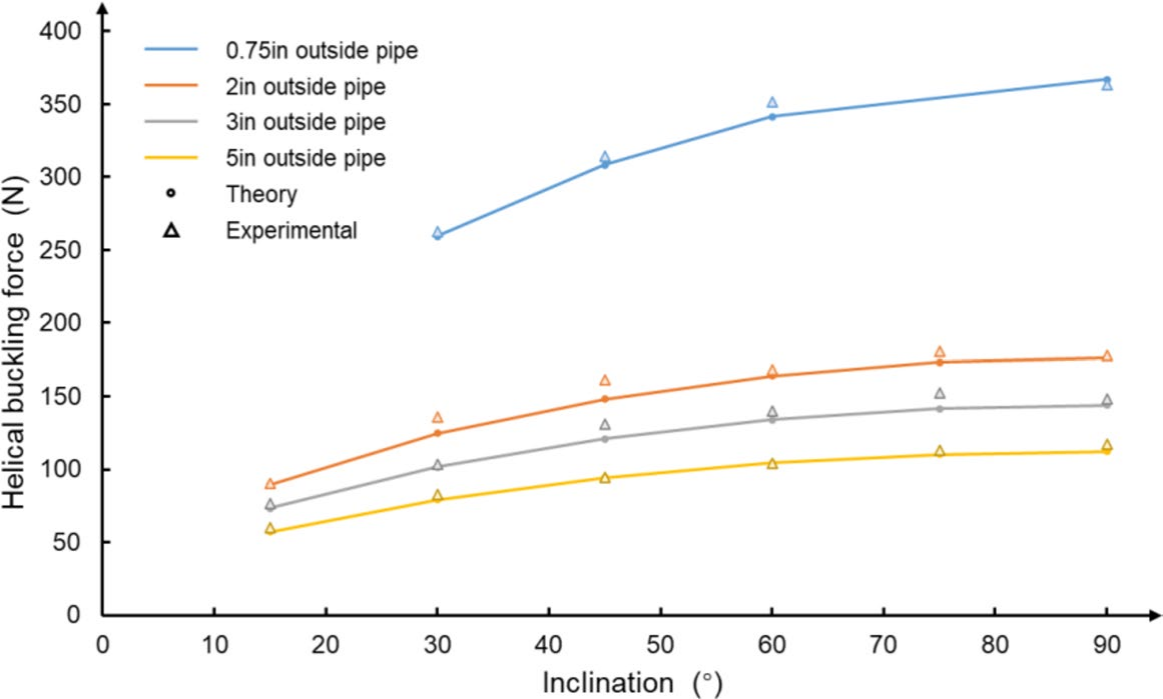
Comparison of calculated and experimental critical loads for helical buckling.

Based on the laboratory experiments reported in Reference [32], the critical helical buckling loads of pipe strings in confined spaces under different inclination angles were presented. The critical values derived from the calculation method adopted in this study show a high degree of consistency with the experimental values, as illustrated in Fig. 4. As illustrated in the figure, the calculated results exhibit a high degree of agreement with the experimental results. The maximum errors occur under the conditions of 2in outer pipe inner diameter with inclination angles of 30° and 40°, reaching 8.24% and 8.14%, respectively. Errors in all other conditions are less than 5%, which demonstrates that the critical buckling criterion adopted in this study is accurate and reliable.

The smooth connection method for unbuckled segments, sinusoidal buckling segments, and helical buckling segments proposed in this study is rooted in the core principle of ensuring the completeness and continuity of geometric configurations. When constructing the entire rod string configuration under three-dimensional wellbore constraints, the primary challenge lies in resolving the geometric description discontinuity between different buckling modes. By introducing smooth continuity conditions, a smooth transition of the displacement field and its first-order derivative is achieved, providing a geometrically self-consistent and differentiable configuration basis for subsequent mechanical analysis. The aforementioned geometric smooth transition is a construction method that ensures the stability of the calculation process and the visual continuity of results. To date, no field cases or full-well rod string tests related to this method have been retrieved in the existing literature.

## Case study

**Fig. 5:**
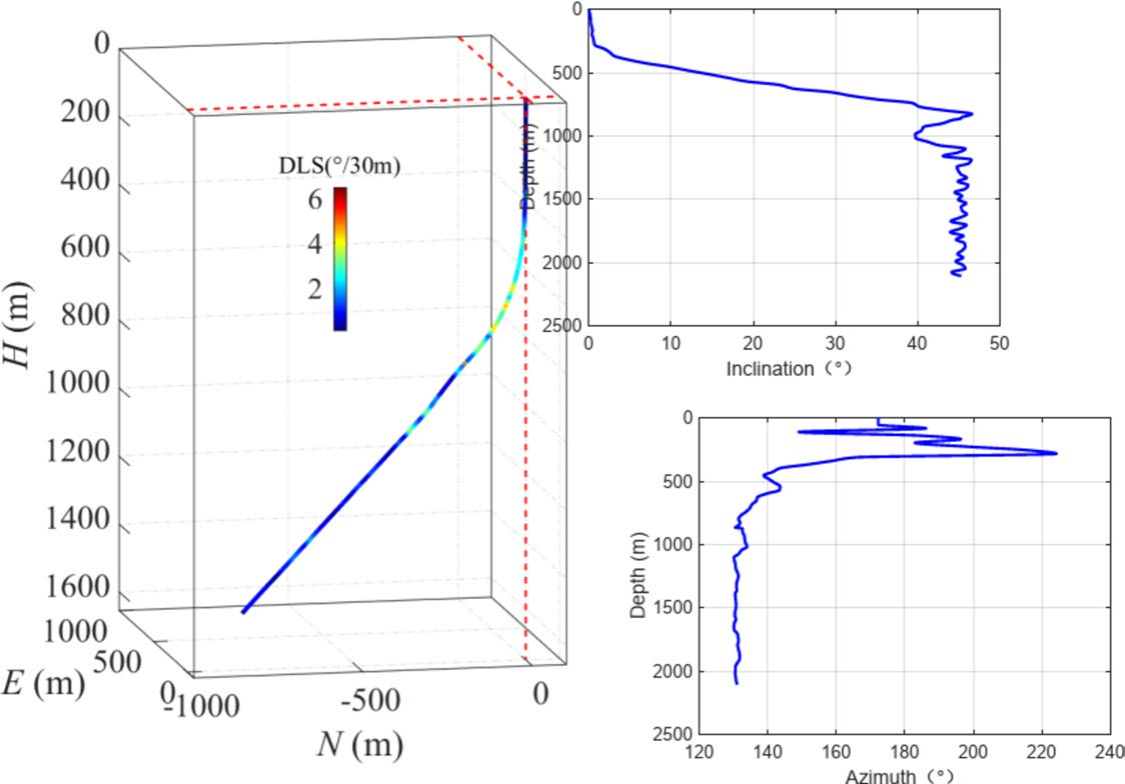
Wellbore trajectory of well J2304.

J2304 is a directional well located in an oilfield in eastern China, with a total depth of 5092.0 m and a pump setting depth of 2000.0 m. The wellbore trajectory is illustrated in Fig. 5, where the color gradient denotes the wellbore curvature. The upper interval of the wellbore corresponds to the vertical section, initiating deviation at the depth of 339.0 m; thereafter, the well inclination angle stabilizes approximately at 45°.

The tubing outer diameter is 73.0 mm with inner diameter of 62.0 mm. The assembly of the sucker rod string is presented in Table 1.

**Table 1:** Assembly of the sucker rod string in well J2304.

Type	Diameter (mm)	Length (m)	Linear Weight (*N*/m)
H-grade	25.0	490.0	40.0
H-grade	22.0	550.0	30.0
H-grade	19.0	960.0	23.8

**Fig. 6:**
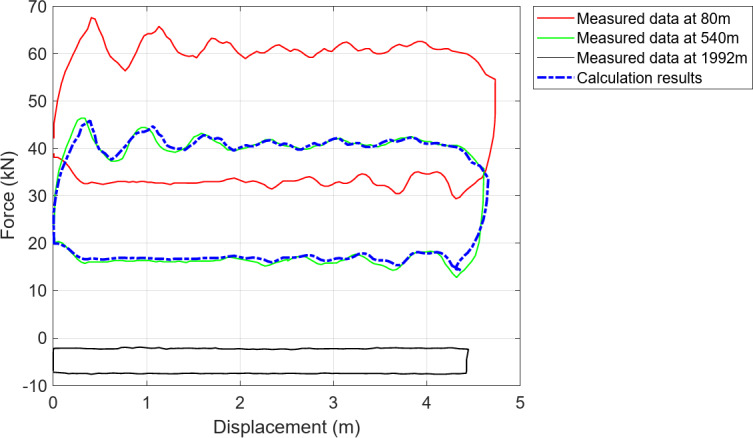
Comparison between calculated and measured dynamometer card.

The outer diameter of the oil well pump is 38.0 mm, the pump length is 9.0 m, the stroke length is 6.0 m, and the stroke frequency is 3.0 min⁻¹. On-site measurements of load-displacement data were taken at three positions: 80.0 m, 540.0 m, and 1992.0 m (see Fig. 6). The well is fully liquid-charged, the sucker rod string is still subjected to the lifting action of the bottom liquid during the upstroke, resulting in a negative load at the bottom end. By adopting the load-displacement curves at the 80 m and the 1992 m as boundary conditions and fitting the friction coefficient, the load-displacement curves of the sucker rod string at any position can be calculated. The measured results at the 540.0 m position are in high agreement with the simulation results, which demonstrates the reliability of the calculation program.

**Fig. 7:**
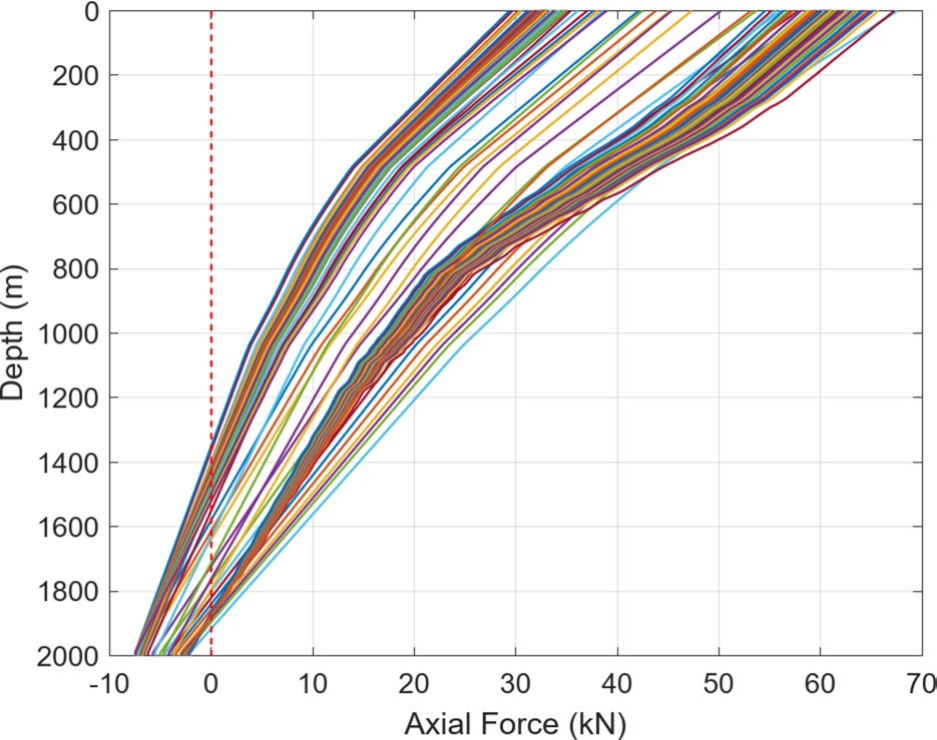
Dynamic axial force of the sucker rod string.

The variation of the axial force of the sucker rod string during its reciprocating motion is illustrated in Fig. 7, curves in different colors correspond to different time instants. It can be observed that during both the upstroke and downstroke of the sucker rod string, the lower section of the string is compressed due to the buoyancy exerted by the wellbore liquid on the pump. During the upstroke and downstroke, the load on the upper section of the sucker rod string exhibits the fluctuations due to the effects of inertial force and friction, whereas the load on the lower section shows minimal fluctuations.

**Fig. 8:**
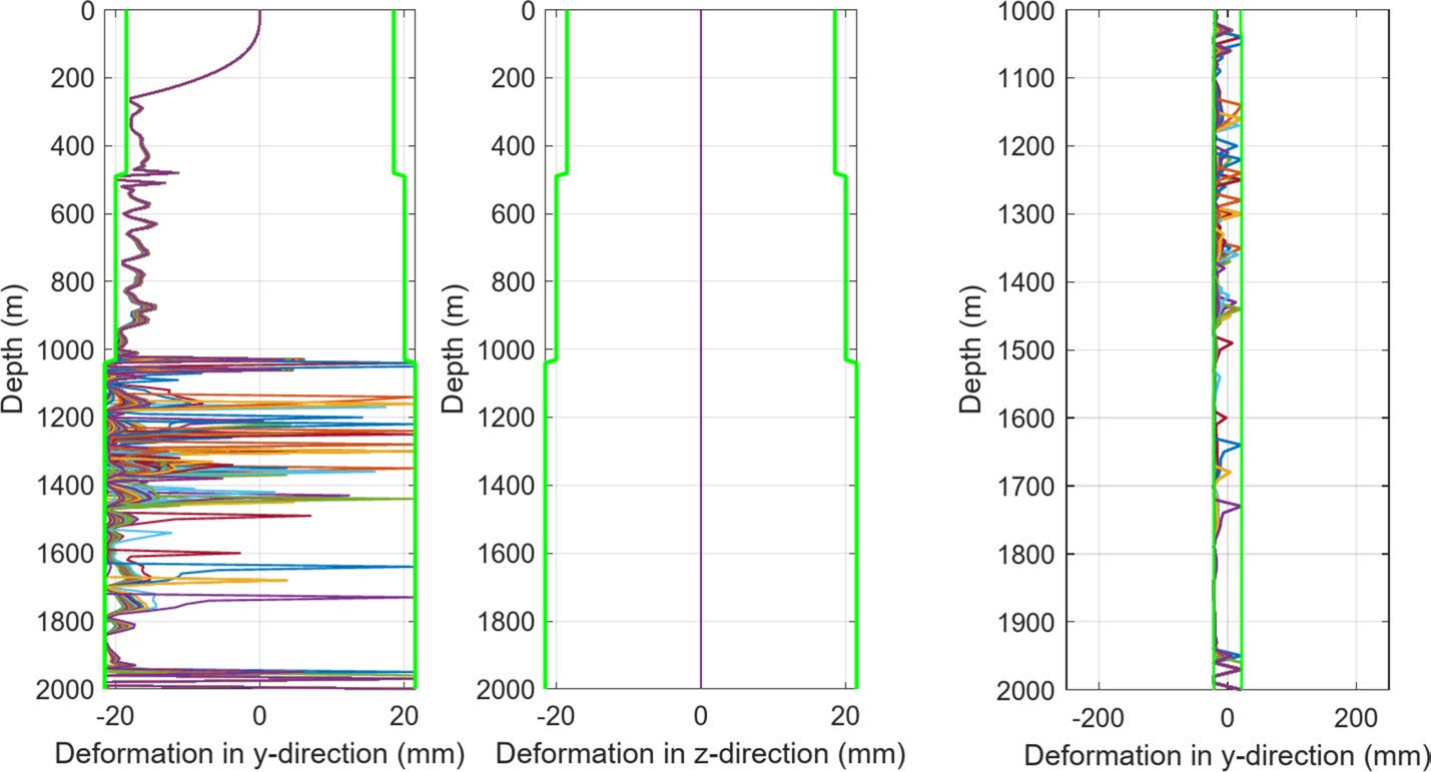
The lateral deformation of the sucker rod string and its magnified view at the Depth Range of 1000–2000 m.

The lateral deformation of the sucker rod string is illustrated in Fig. 8. It can be observed that owing to the small inclination in the upper section of the well, the sucker rod string does not contact with the tubing under the axial tensile force acting on its upper section. As well deviation increases and the tubing–rod annular clearance widens (the green curve in the figure indicates the clearance threshold), the sucker rod string undergoes significant lateral deformation. Owing to the coupling effect of longitudinal-torsional-lateral interactions, as well as the influences of the reciprocating motion inertia of the sucker rod string and variations in wellbore trajectory, the lateral contact behavior between the sucker rod string and the tubing demonstrates complex time-dependent characteristics, particularly at the third-stage rod string with smaller dimensions. Furthermore, due to gravitational effects, contact with the lower tubing wall remains dominant. Given that the azimuth of the wellbore trajectory remains essentially constant, meaning the well can be approximated as a two-dimensional planar well, the deformation of the sucker rod string in the binormal direction (z-direction) is negligible.

**Fig. 9:**
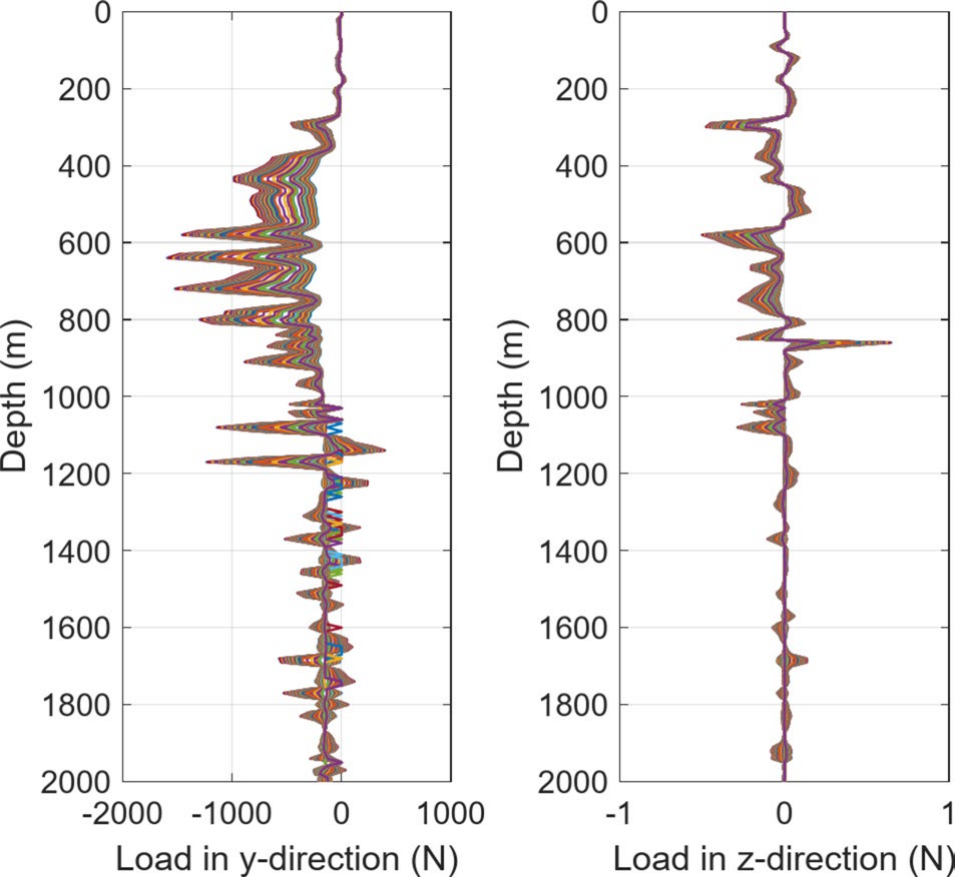
The lateral load of the sucker rod string.

The lateral load of the sucker rod string is illustrated in Fig. 9. As depicted in Fig. 9, the lateral load of the sucker rod string is predominantly distributed along the y-direction, i.e., the well deviation direction, with the gravitational component acting as the dominant contributor. In the curved well section, the load of the sucker rod string in the y-direction exhibits significant variations due to the coupling effect of axial tension, contact force, and gravity component. Additionally, owing to the two-dimensional planar character of the wellbore trajectory, the load in the z-direction is negligible.

**Fig. 10:**
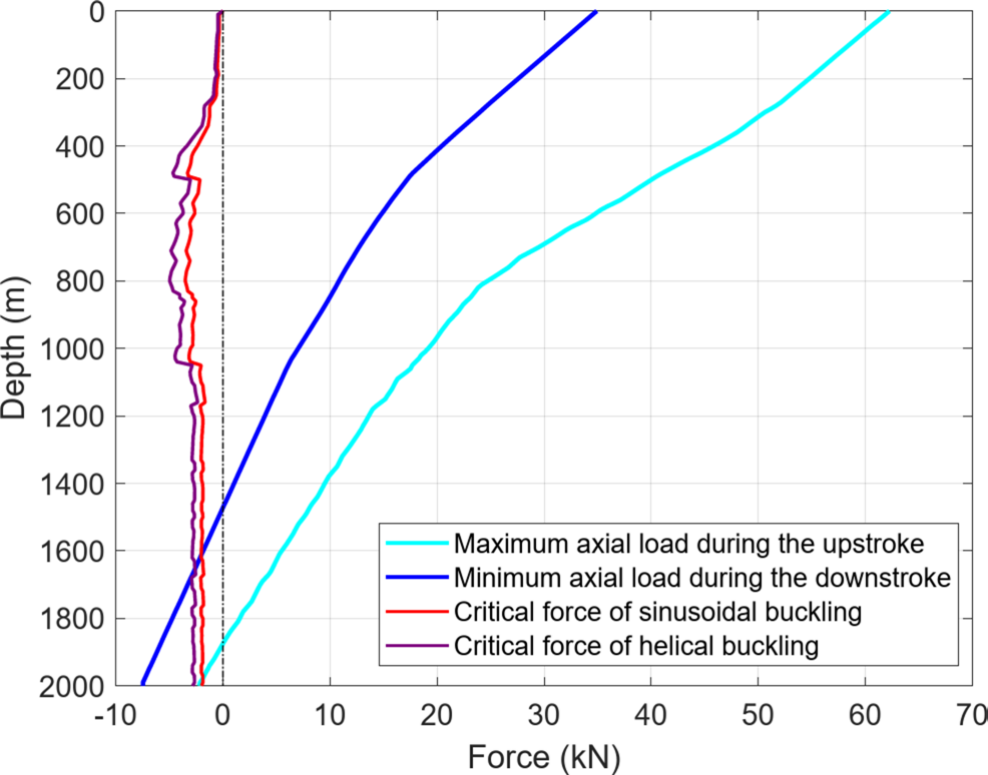
Critical buckling assessment of the sucker rod string.

In practice, if the axial load exceeds the critical buckling force, buckling will initiate, and the deformation will rapidly transition to a sinusoidal or helical configuration. The curves of the maximum and minimum axial forces during the motion of the sucker rod string, along with the critical load for sinusoidal and helical buckling are presented in Fig. 10.

In Fig. 10, it can be observed that when the sucker rod string is subjected to the maximum tensile force during the upstroke, the pressure at the bottom end of the string is approximately − 2.0 kN, which is close to the critical force for sinusoidal buckling. This suggests that a sinusoidal buckling state may exist at the lower end of the string. During the downstroke, the minimum load at the bottom end of the sucker rod string is approximately − 7.50 kN, which has significantly exceeded the critical forces for both sinusoidal and helical buckling. At this stage, the sucker rod string exhibits coexisting unbuckled, sinusoidal buckling, and helical buckling states. It should be noted that although buckling induces a minor perturbation to the axial force distribution along the sucker rod string, this effect is excluded from the present analysis to maintain focus on the three-dimensional spatial configuration of the entire sucker rod string.

**Fig. 11:**
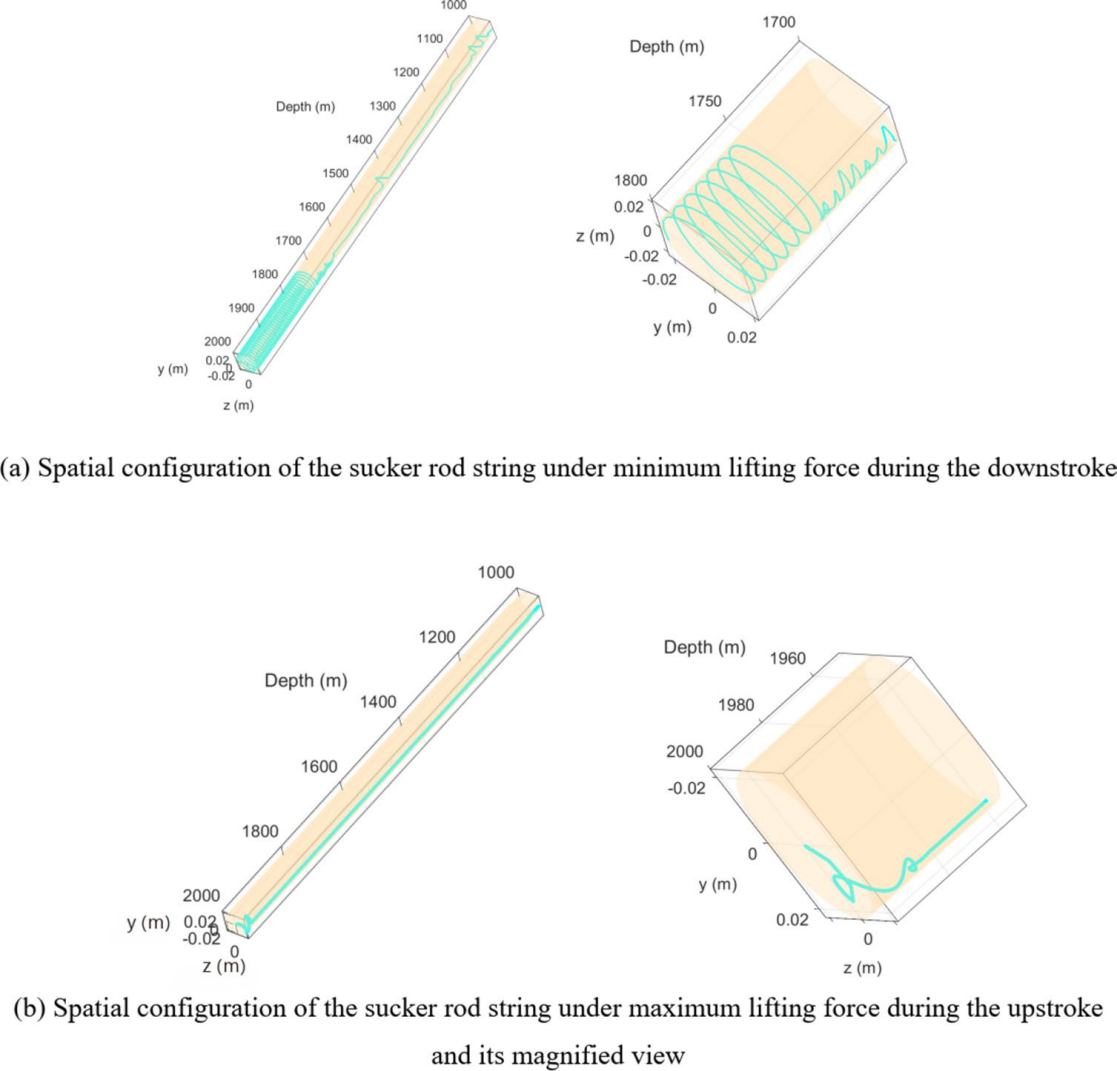
Spatial configurations of the sucker rod string at different time instants.

**Fig. 12:**
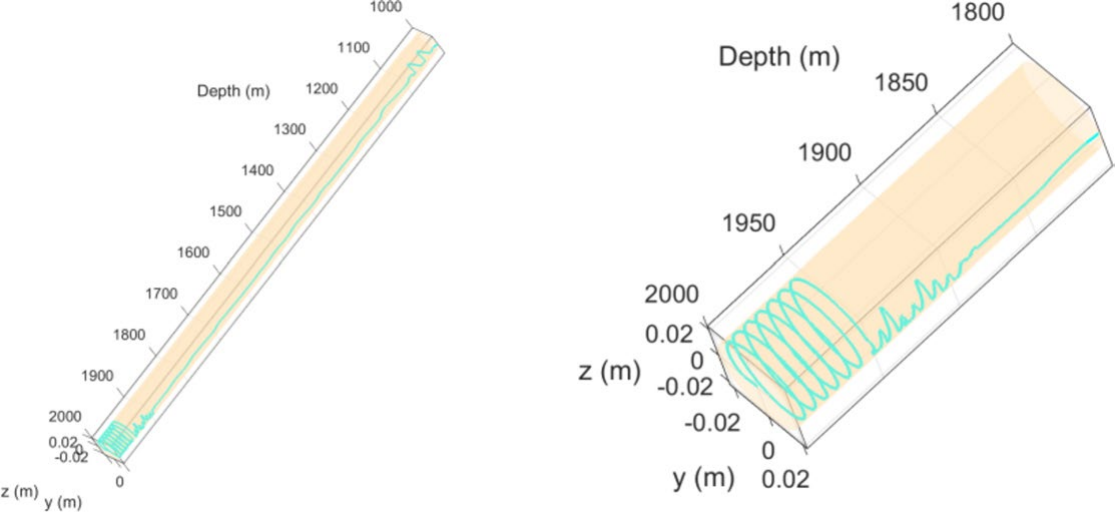
Spatial configurations of the sucker rod string at the initial stage of the downstroke.

Based on the spatial configuration construction method proposed in Sect.  2.3, the dynamic spatial configuration of the sucker rod string within the tubing at different moments can be obtained, as shown in Fig. 11 (with a 1200-fold horizontal magnification). When the sucker rod string is subjected to the maximum lifting force during the upstroke, the bottom end of the sucker rod exhibits a sinusoidal-like deformation. When the bottom end of the sucker rod is subjected to the maximum compressive load during the downstroke, the three modes of the sucker rod string coexist. And approximately 260.0 m of the lower segment undergoes helical buckling, while around 60.0 m exhibits sinusoidal buckling. Furthermore, the pitch of the helical buckling at the bottom is inversely proportional to the axial compressive pressure. In actual operation, the spatial configuration of the sucker rod string evolves continuously between the two limiting configurations depicted in Figs. 11(a) and 11(b) throughout its axial reciprocating motion. For instance, at the initial stage of the downstroke, its spatial configuration is illustrated in Fig. 12.

The load variation experienced by the sucker rod string during its reciprocating motion is a critical factor governing its spatial buckling configuration. Notably, the occurrence of sinusoidal buckling, helical buckling, and unbuckled states for a rod constrained within tubing has been qualitatively observed in the experiments reported in Refs.^26,31,32^ and related studies. Although these experimental observations do not directly replicate the full‑well scale configuration, they substantiate the existence of such buckling modes and provide essential qualitative support for the full‑well configurations of the entire sucker rod string obtained in the present work.

## Conclusions

Drawing on the three-dimensional dynamic simulation results of the sucker rod string and the critical conditions for buckling stability, this study introduces the continuity and smoothness conditions of rod deformation to construct the spatial configuration of the entire sucker rod string during its movement within the tubing. This method enables an accurate reflection of the actual downhole operating state of the sucker rod string, thereby laying a theoretical foundation for subsequent assessment of its eccentric wear behavior and implementation of anti-eccentric wear countermeasures.

It should be noted that while this study has incorporated the influence of dynamic friction on the sucker rod string load, the in-depth mechanism by which it affects the spatial configuration of the sucker rod string has not yet been systematically elucidated. Additionally, the effect of the variable cross-section characteristics of the sucker rod string on its spatial configuration requires further analysis. These factors are significant for determining the rotational direction of helical buckling, and subsequent research will be conducted to explore these issues in depth.

## Data Availability

The datasets used and/or analysed during the current study available from the corresponding author on reasonable request.
